# Evaluation of the Effect of a Mobile Application on Glycated Hemoglobin in Older Adults with Type 2 Diabetes Mellitus—Protocol of a Randomized Clinical Trial

**DOI:** 10.3390/nu16193360

**Published:** 2024-10-03

**Authors:** Raíza Rana de Souza Lima Trombini, Rafaella Dusi, Alayne Larissa Martins Pereira, Renata Puppin Zandonadi, Marina Morato Stival, Verônica Cortez Ginani, Silvana Schwerz Funghetto

**Affiliations:** 1 Postgraduate Program in Health Sciences and Technologies, University of Brasília, Campus Universitario Ceilândia, Brasília 72220-275, Brazil; pereira.alayne@aluno.unb.br (A.L.M.P.); marinamorato@unb.br (M.M.S.); silvanasf@unb.br (S.S.F.); 2 Department of Nutrition, Faculty of Health Sciences, University of Brasília, Campus Universitario Darcy Ribeiro, Brasília 70910-900, Brazil; rafaella.dusi@aluno.unb.br (R.D.); vcginani@unb.br (V.C.G.)

**Keywords:** healthy aging, Brazilian older adults, mobile health interventions, type 2 diabetes mellitus, self-management, educational technology, prevention

## Abstract

Background: Digital educational technologies in health have been an important instrument for promoting learning, self-care, self-esteem, and security regarding prevention and health promotion actions that lead to changes in behavior, mainly for non-communicable disease patients, such as type 2 Diabetes Mellitus (DM 2). Objective: This study aimed to describe a protocol for evaluating the effect of an app for cell phones and tablets on the blood glucose of older adults with DM 2. Methods: The protocol will be used to compare the effectiveness of an application for mobile devices concerning the educational booklet in reducing Glycated Hemoglobin in older adults with DM 2 in Primary Health Care. This protocol is part of a Randomized Clinical Trial project entitled Effectiveness of a Mobile Device Application on Glycated Hemoglobin in Elderly People with Type 2 Diabetes Mellitus: a Randomized Clinical Trial. Results: The protocol was structured in the following phases: (i) sample calculation, (ii) invitation to participate in the study according to the eligibility criteria; (iii) participant registration; (iv) randomization and allocation of participants into groups (double blinding); (v) application of the intervention; (vi) post-intervention procedures (post-test); (vii) data analysis. Conclusion: It is expected that encouraging studies on the impact of a mobile application will improve and enhance health education focused on self-care for older adults with DM 2, potentially influencing the local health system by reducing hospitalizations due to conditions that are sensitive to primary care, since health promotion and prevention of DM-related illnesses will be the main focus of the application and booklet developed.

## 1. Introduction

Currently, a change in fertility and mortality rates has been emerging from the increase in longevity expectations and the number of older adults around the world.

The aging of the world’s population is happening at an accelerated pace, and as a result, global life expectancy has increased significantly. In 2020, there were approximately 1 billion people aged 60 or over, with this number expected to rise to 1.4 billion by 2030 and to reach 2.1 billion by 2050. In 1990, the average life expectancy was 64 years, but by 2019, it had risen to 73 years. In this sense, coordinated and rapid actions are essential to support healthy aging, especially in low- and middle-income countries, since 80% of the world’s elderly population will be concentrated there by 2050 [1,2].

In Brazil, older adults represented over 14% of the total population at the end of the last decade, and it is estimated that in 2050, it will increase by about 50% [3,4,5,6]. The aging process generally comprises complex changes linked to the individual’s biological, psychological, and social components, which gradually interfere with behavior and social interactions. People in the aging process sometimes re-signify goals and activities, optimizing and prioritizing their existing capabilities through new practices and technologies to remedy the loss of some skills and find other ways to perform tasks. Reframing and adapting to maintain autonomy in priority tasks are part of healthy aging, as shown in the World Report on Aging and Health [1,2].

In 2015, the 193 Member States of the United Nations (UN) came together, aiming to put the world on a sustainable path. Then, the 2030 Agenda was established to implement the Sustainable Development Goals (SDG). Health and well-being are the central points of the 3rd SDG, which aims to guarantee universal and quality access for all ages. Among the goals of the 3rd SDG is the reduction, by one-third, of premature mortality from non-communicable diseases, such as chronic non-communicable diseases (NCDs), through prevention and treatment, achieving universal coverage, and through access to essential quality healthcare services [7,8].

The 2030 Agenda highlights that the dissemination of information and communication technologies (ICT) and global interconnectivity have a great ability to accelerate human progress, eliminate digital exclusion and develop the spread of knowledge, health education and health care to the largest number of people, especially those who are out of reach of or inaccessible to traditional care [6,7,8].

It is recognized worldwide that ICT point to new opportunities for the strategic and innovative use of cutting-edge digital information. Professional training and education for the general population are essential to ensure that an additional 1 billion people benefit from universal health coverage and well-being, including during global health emergencies [9].

In this context, eHealth, which concerns the safe, economic, ethical, equitable, and sustainable use of ICT in support of health—such as in health services, health surveillance, health literacy and education, and knowledge—is of fundamental importance to achieving the goals of the 3rd MDG [9].

eHealth is included in the Digital Health strategy, which encompasses the use of other digital technologies for health, such as the Internet of Things, artificial intelligence, big data, and robotics. These digital technologies enable data storage and tools that support remote data capture and exchange, as well as information sharing. This sharing can create a chain of care with proven potential to improve health outcomes, including diagnosis, treatment, digital therapeutics, clinical trials, self-management of care, and person-centered care [9,10].

To strengthen the digital health strategy, the WHO entered into a partnership with the International Telecommunications Union (ITU) through the “Be He@lthy, Be Mobile” initiative, supporting the expansion of mobile health technology (mHealth) in health systems services to help face NCDs and support healthy aging. mHealth is defined as medical and public health practice supported by mobile devices, such as cell phones, patient monitoring devices, personal digital assistants and other wireless devices [9,10]. From mHealth, mHealth for aging (or mAgeing) emerged as a new program whose central objective is to help older adults, in the case of Brazil, people over 60 years of age, to maintain functional capacity and live as independently and healthily as possible through evidence-based self-management and self-care interventions, complementing routine care already implemented by healthcare teams [9,10].

The findings of the 10th edition of the International Diabetes Federation (IDF) showed that DM is one of the fastest-growing global health emergencies of the 21st century [9]. In 2021, around 537 million people had this condition, with projections that this number will reach 643 million by 2030, and 783 million by 2045. According to the IDF, in 2021, altered fasting glucose levels worldwide were increasingly present with advancing age in adults, reaching a peak of 8.4% in people aged 60–64. Regarding the prevalence of diabetes in people aged 65–79 in Brazil, a prevalence of 19.9% was identified, and in those over 75 years old, it was 21.1% [11].

Among DM individuals, 50 to 80% have a deficit related to knowledge about the disease and self-care skills necessary for treatment to be effective and disease prevention to be achieved. This data corroborates the results found in a study by Borba et al. (2019) that out of the 202 elderly participants, 77.7% had insufficient knowledge about DM and, regarding attitude, 85.6% had a negative psychological adjustment concerning diabetes [12]. In this sense, empowerment favors the glycemic control process, which results from autonomy and co-responsibility for treatment [13,14].

An Australian study that aimed to identify what would be a “perfect application” for DM showed that the practical aspects of self-management of the disease, the guidelines regarding the measures to be taken, reminders and alarms, as well as the approach to topics for managing stress and negative emotions were the main topics lacking guidance in an application [15].

A study by Kebede and Pischke [16] aimed to identify popular diabetes apps with functions that encourage self-management, such as monitoring blood glucose, physical activity and meal planning, and investigating the association of the use of these types of apps and other factors with cumulative self-care behavior. The study included 1682 participants (1052 with type 1 DM (DM 1) and 632 with type 2 (DM 2), of which more than half, 549 (52.2%), and one third, 210 (33.3%), of the respondents with DM 1 and DM 2, respectively, reported using diabetes self-management apps. Although participants with DM 1 reported using self-management apps more than those with DM 2, there was a greater increase in the cumulative score for self-care in participants with DM 2. It is worth noting that the number of people with DM 2over 60 years of age was three times higher than with DM 1 in the same age group, which explains the greater use of applications by people with DM 1, who are, for the most part, younger and more familiar with digital technologies [16].

Another study, conducted by Gerber et al. (2024) [17], developed and evaluated the feasibility of an eHealth diabetes prevention program adapted for older adults through a randomized clinical trial. The results showed that appropriate weight loss goals, flexible dietary composition, and accessible interventions for learning through online classrooms and fitness monitoring technology were effective in reducing weight in older adults. Participants in the intervention group had an average weight loss change of 9.5% compared to the control group, which gained 2.4% of their body weight. In this context, it was possible to observe that eHealth and mHealth interventions, which were previously considered inaccessible and ineffective for older adults, are new accessible treatment alternatives that promote active and healthy aging [17].

Corroborating the aforementioned study, in 2020, Whitehouse investigated the feasibility of diabetes self-management education and support (DSMES) via telehealth for DM 2 older adults after hospital discharge. The study showed that, 30 days after post-discharge follow-up, through interventions via a tablet with remote online meetings, participants who completed the intervention increased their diabetes knowledge scores, in addition to a 1.1% decrease in glycated hemoglobin values. Additionally, it was found that there were no hospital readmissions for any patient during the intervention period, which generated satisfaction among participants, who described the program as useful [18].

In this sense, for the DM older adult population to have a healthy aging process, they must be increasingly welcomed and included in the contemporary technological revolution. To this end, digital educational technologies in health have been an important instrument that aims to promote learning, self-care, self-esteem, and security regarding prevention and health promotion actions that lead to changes in behavior [19,20,21,22].

However, for older people to be welcomed into this technological revolution, usability must be one of the main properties of software. According to the Brazilian Association of Technical Standards (ABNT), in the International Organization for Standardization (ISO)/International Electrotechnical Commission (ISO/IEC 25010:2011), usability is defined as “the ability of the software product to be understood, learned, operated and attractive to the user, when used under specified conditions”. In this sense, applications with high usability are more intuitive, which leads to greater user satisfaction and, consequently, greater interaction with the interfaces offered and retention of the educational content of the application [23].

In a systematic review published in 2023, which aimed to develop a set of guidelines aimed at optimizing the design and usability of mobile applications for older adults, twenty-seven guidelines were identified, of which two stood out in 15 of the 40 studies included in the review, namely: simplifying the design due to the possible cognitive changes present in this population and increasing the size and distance between interactive controls, such as buttons and form inputs, to avoid accidental touches on icons [24].

Still regarding the guidelines, the others were organized into five categories, such as: “Help and Training”, which are focused on creating tutorials on how to navigate the application; “Navigation”, which should have as a characteristic the reduction in the number of alternative paths to perform a task, focusing on the current action; “Visual Design”, such as reducing the number of elements and options available on the screen and making them familiar to the elderly person, with a strong contrast between the color of the text and the color of the background, as well as the use of large fonts; Adequate “Cognitive Load” through the use of simple, short, and unambiguous language, according to the context in which the person is inserted, and finally, “Interaction”, advocating less use of the keyboard and reduced sensitivity to touch [24].

A usability study sought to identify barriers and facilitators, based on design, for completing health surveys on a mobile application among older adults participating in the Framingham Heart Study and a diverse hospital environment. Corroborating the guidelines previously mentioned, the research participants verbalized a preference for large font sizes and friendlier colors. Regarding cognitive load, older adults had no difficulty with the questions presented in the application, unless they presented long instructions. In this sense, the application was characterized by the majority as “easy and fun” and that through it they were able to understand the importance of using it to raise awareness about their own health. As a suggestion, the participants reported the importance of using voice instructions and animated tutorials [24,25].

Other studies report that older people are more likely to adopt mobile device applications when they perceive them as useful for their own health and when they feel empowered to use them through training and tutorials, which leads to greater engagement in the use of technology and a lower error rate during navigation [26,27,28].

Usability is even more important when a new digital technological tool is developed for people over 60 years old due to the digital literacy barriers they face, such as cognitive decline, decreased visual acuity and lack of familiarity with digital interfaces. Therefore, application design must incorporate usability principles, such as larger fonts that make text readable, simplified navigation, large icons, color contrast, and audio features to make technology more accessible [29].

Therefore, this study aimed to describe a protocol for evaluating the effect of an app for cell phones and tablets on the blood glucose of older adults with DM 2. The protocol aims to compare the effectiveness of an application for mobile devices concerning the educational booklet in reducing Glycated Hemoglobin in older adults with DM 2 in Primary Health Care (PHC).

The primary hypothesis of the study in which this protocol will be applied is that older adults with DM 2 who use the mobile application will have a greater reduction in glycated hemoglobin, and will have a higher level of knowledge, self-care and self-efficacy compared to older adults who use the educational booklet.

## 2. Protocol Description

### 2.1. Study Design

A Randomized Clinical Trial (RCT) will be performed, in which the intervention group will use an application for mobile devices, and the control group will have access to a printed booklet, both with content related to the care of older adults with DM 2. The application for mobile devices developed in a previous study will be applied in this RCT and has the following interfaces: user profile (age, weight, height, Blood Pressure, Blood Glucose, waist circumference), food diary, guidelines and physical exercise plan, record of medications in use, leisure and games (crosswords and word searches) to fix content [30,31].

For the appropriate design of the RCT, the Consolidated Standards of Reporting Trials of Electronic and Mobile Health Applications and online TeleHealth (CONSORT-EHEALTH) and the SQUIRE 2.0 guidelines were used as a basis for the development of the study, considering that these instruments have been recommended by the scientific community, to ensure the validity of the study and applicability of the results [32,33].

### 2.2. Sample Calculation

Considering a α ≤ 5% risk for Type I error, β ≤ 20% risk for Type II error and a power of 80%, estimating an expected reduction after the pre- and post-intervention periods, referring to the use of the application, of 1.24 mg/dL of glycated hemoglobin with a standard deviation of 1.48 mg/dL, an effect size of 0.838 is obtained. In this way, the sample the protocol will be applied to consists of at least 17 older adults with pre-diabetes or DM 2 per group, considering subdivision by extracts and segment losses.

### 2.3. Study Location and Participants

The RCT will be conducted in a Basic Health Unit (BHU) in Ceilândia, Federal District/Brazil, for 3 months by the Health, Care and Aging Research Group (GPeSEn) team at the University of Brasília. This study is part of the GENIOS Project, which develops digital technologies for the self-management of older adults’ health.

The older adults who will participate in the study will be invited through an active search in the Hypertension and Diabetes groups and in routine consultations at the BHU. After applying the inclusion and exclusion criteria (Table 1), they will sign the Free and Informed Consent Form.

### 2.4. Blinding

As an RCT that uses two educational technologies as an intervention, participants will be blinded only regarding the research hypotheses and not regarding the two interventions used. Therefore, blinding regarding randomization, the type of educational technology, and data analysis, pre- and post-intervention, will be applied to the leading researcher and the statistician. The entire process will be conducted by a previously trained and qualified participant from the research group. Blinding will prevent the leading researcher’s biases and prevent differentiated attention to the intervention group.

### 2.5. Application of the Pre-Test

Before distributing the printed booklet to the control group and installing the application on the smartphones of members of the intervention group, the older people who meet the research criteria and sign the informed consent form will respond to a socio-demographic questionnaire, the Diabetes Knowledge Scale (DKN-A), the Diabetes Self-Care Activities Questionnaire (QAD), the Diabetes Self-Efficacy Scale—Short Version (EAD-VC), the Eating Competence Questionnaire, the Determine your Nutrition Questionnaire (DNH) and VIGITEL.

In addition to the instruments that will allow verifying the baseline state of knowledge, self-care, self-efficacy regarding DM and nutritional behaviors of the participants, biochemical blood and urine tests will also be collected, such as: Glycated hemoglobin, fasting blood glucose, lipid profile, serum creatinine and type 1 urine test, according to the IV Brazilian Guideline on Dyslipidemias and Prevention of Atherosclerosis and the Brazilian Diabetes Society.

### 2.6. Randomization and Allocation

As the expected primary outcome will be the reduction in glycated hemoglobin after 3 months of intervention, after obtaining the results of this biochemical parameter in the pre-test period, participants will be randomized and allocated into the intervention and control groups in a stratified manner using the Microsoft Excel program, considering the glycated hemoglobin baseline, so that the values of this parameter are similar in both groups and the sample is homogeneous.

### 2.7. Application of the Intervention

According to the American Association of Diabetes Educators [31], it is necessary to establish a basic education program on diabetes that covers several topics, encouraging self-care and improving quality of life. The main topics of this program include a healthy eating plan, controlling glucose levels, practicing regular physical activity, adhering to medication treatment, and encouraging behaviors that aim to reduce the risk of complications. In this context, the application was created with the aforementioned topics, including weekly notifications of educational and motivational messages validated in a previous study [31] about healthy eating as well as foot care, audios for guided imagery and meditation, healthy recipes aimed at people with diabetes, and games such as “Crosswords” and “Word Search” to reinforce the main topics for diabetes control. The educational booklet was created with the same content as the app, including games such as “Crosswords”, “Word Search”, and “What is the Intruder Food?” [30].

To prepare the content of the topics covered in these educational tools, a bibliographic review was conducted, covering the main Brazilian and global references on the primary care measures aimed at elderly people with diabetes [36,37,38,39,40,41,42,43].

A team of scholarship holders specializing in programming and digital game design was selected to develop the app and the booklet, as well as the images inserted in them, through the University of Brasília’s Notice 01/2022: “Selection of Scholarship Holder for the Research Project Technologies for Health Management and Self-Care for the Elderly—GENIIO-S Project”.

The interventions that will be implemented in this study were developed according to the Contextualized Instructional Design (CID) method. CID refers to a method that aims to describe the planning, development and application of contextualized educational content, and is also used for developing software in the health area CID follows five stages for the systematic construction of educational technologies, namely: analysis, design, development, implementation and evaluation [44]. The CID method was chosen because it meets the work style of the interdisciplinary team that was part of the development of the educational tools developed.

In the Design and Development phases, the following interfaces and sections of the application and booklet were developed, as shown in Table 2.

It is worth noting that both the mobile app and the educational booklet were previously validated by researchers using experts and DM 2 older adults groups [30,31].

During the execution of the clinical trial, older adults in the intervention group (IG) will receive 30–60 min of training on how to use the application, as well as having it installed on their mobile devices by the project team. They will be informed about the continuity of the application surveyed by telephone/messaging application, every fortnight, for three months (intervention time), so that possible doubts about the use of the application can be clarified.

The participants in the control group (CG) will receive an educational booklet previously developed and validated by experts, and its content will address the same themes as the application. At this meeting, the content of the booklet will be presented, in general, for 30–60 min. After the presentation, the researcher will inform the participants that they will take the booklet with them to read, for three months (intervention time), regarding self-care with DM 2.

Regarding the exercises proposed in the application, the adaptation phase will be carried out, which consists of performing the exercises that comprise the aerobic and resistance exercise protocol proposed in the application. Thus, older adults will attend the UBS for two weeks before the intervention phase, on two non-consecutive days a week.

During the 3-month intervention, the following procedures will be carried out:

- Month 1—resistance exercise protocol twice a week on non-consecutive days, consisting of two series for each exercise and a 30-min walk twice a week.
- Month 2—resistance exercise protocol twice a week on non-consecutive days, consisting of three series for each exercise and a 30-min walk three times a week.
- Month 3—resistance exercise protocol twice a week on non-consecutive days, consisting of five series of each exercise and a 30-min walk five times a week.

Both groups (IG and CG) will perform the exercise intervention. All patients will receive the usual treatment (routine consultations with the Unit’s professionals) provided by the BHU, according to their individual needs, which is why the educational intervention should be considered an additional service.

### 2.8. Post-Intervention Procedures (Post-Test)

After 3 months of using the app or educational booklet and the resistance and aerobic exercise protocol, older adults who are part of the GI will answer a questionnaire based on the System Usability Scale (SUS). The SUS is a questionnaire used to evaluate the usability of systems, products, or services. Comprising 10 items, the SUS includes the following statements: 1. “I think I would like to use this system frequently”; 2. “I found the system unnecessarily complex”; 3. “I thought the system was easy to use”; 4. “I think I would need the support of a technical person to be able to use this system”; 5. “I found the various functions in this system were well integrated”; 6. “I thought there was too much inconsistency in this system”; 7. “I would imagine that most people would learn to use this system very quickly”; 8. “I found the system very cumbersome to use”; 9. “I felt very confident using the system”; and 10. “I needed to learn a lot of things before I could get going with this system”) [45].

When comparing the SUS to other instruments used to assess usability, such as the User Interaction Satisfaction Questionnaire (QUIS) and the Usability Metric for User Experience (UMUX), it is clear that the SUS stands out in terms of versatility, as it can be applied to various systems, such as websites, mobile applications, and medical systems, in addition to being simple and easy to apply. The UMUX, despite being a concise instrument like the SUS, differs in that it focuses on the user experience, while the QUIS is more complex, evaluating user interaction through 50 questions [46,47].

**Figure 1:**
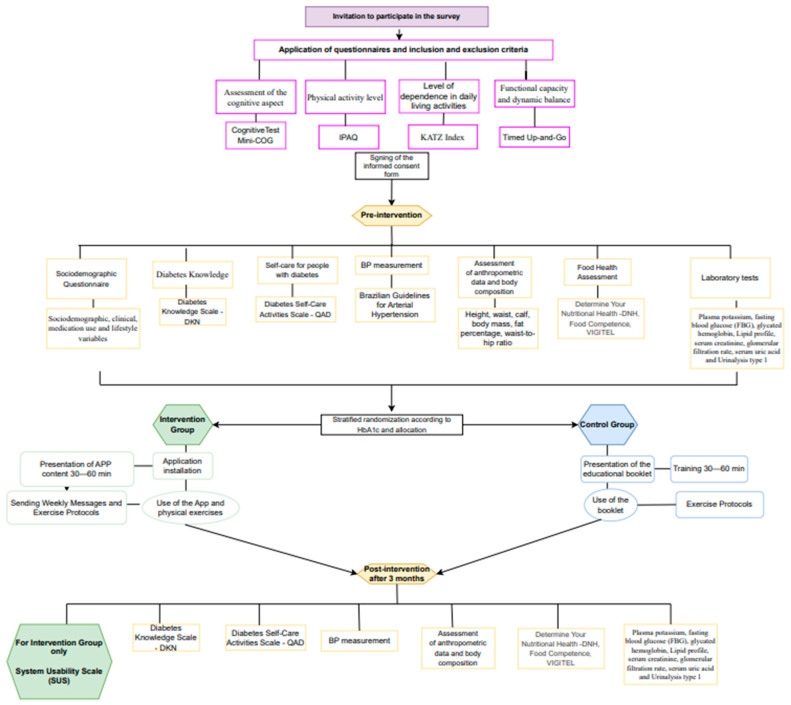
Protocol of the clinical trial stages.

Both groups repeat the assessments and laboratory tests carried out in the pre-test to compare the measurements before and after the intervention between and within groups. In this way, it will be possible to evaluate the effect of using the application on older adults. After protocol implementation, both the GI and the GC will have access to the application and the printed booklet. The stages of the clinical trial protocol are described in Figure 1.

### 2.9. Data Analysis

For data analysis, the Package for the Social Sciences (SPSS^®^) version 20.0 software will be used, in which a database will be built. An initial analysis will be performed to identify typing and classification errors, making corrections when necessary. Statistical tests will be performed as needed. Values of *p* < 0.05 will be considered statistically significant.

Regarding the evaluated and analyzed variables, the nominal qualitative variables are: sex, marital status, place of residence, use of continuous medications, comorbidities, alcoholism, smoking, physical activity, and the presence of cells such as leukocytes and erythrocytes, bacteria, and color in the type 1 urine test. As for the ordinal qualitative variables, the following are considered: education level, urine appearance (e.g., clear, cloudy), and color intensity (e.g., light, dark) in the type 1 urine test, perception of health status, knowledge about diabetes mellitus (DM), level of self-care in DM, and level of self-efficacy in DM. The continuous quantitative variables include: income level, age, weight, height, glycated hemoglobin, fasting glucose, total cholesterol, triglycerides, HDL, LDL, serum creatinine, and type 1 urine test (pH, density, glucose, proteins).

To apply the SUS, the questionnaire is distributed to users after interacting with the system. Responses are scored from 1 to 5, with 1 subtracted from the scores of odd-numbered questions and the scores of even-numbered questions subtracted from 5. This calculation normalizes the scores to a scale from 0 to 4 points. The normalized scores are then summed and multiplied by 2.5, resulting in a final score ranging from 0 to 100. Scores above 68 are considered above average, while scores below indicate a need for improvement. The SUS is valued for its simplicity, flexibility, and ability to compare different systems or versions) [45].

## 3. Final Considerations

The growth in life expectancy worldwide, nationally and, especially, in the Brazilian Federal District (DF), which has the highest Municipal Human Development Index (MHDI) in Brazil (0.850), classified as very high, demands care, since longevity brings with it health conditions that should be the target of investment to control diseases. The MHDI encompasses aspects related to income, education and longevity, and the DF’s “very high” MHDI brings responsibilities to the government and the local scientific community, having as a common goal the increase and maintenance of the quality of life of elderly people with DM.

In view of this, the development and adoption of new technologies within the scope of mHealth for the care of the elderly population will allow the popularization of technology in favor of public health in the Federal District (DF). Thus, in addition to the high-impact scientific-technological production that will strengthen multidisciplinary clinical practice, it will also provide the local community with an application and an educational booklet designed according to the needs of older adults with DM 2 living in the city, especially those who are users of the Unified Health System (SUS).

Therefore, this protocol will help us to conduct studies seeking to innovate the care of older people with technologies that improve health education focused on self-care, will directly and indirectly influence the physical overload of the local health system, and may even reduce hospitalizations for conditions sensitive to primary care, since health promotion and prevention of NCDs will be the main focus of the mHealth application developed for older adults with DM 2.

As for limitations, we highlight that since this protocol has not yet been implemented, adaptations can occur during the study. In addition, the target population of older adults who, despite having and using smartphones, are still in the process of digital inclusion, which may lead to resistance to effectively using the application during the 3 months of the study.

It is expected that, after the full implementation of this protocol, the application will be improved based on considerations from the study population so that it can be progressively implemented in the routine of other older adults with DM in the public health system of the Federal District.

Based on this research, other large-scale studies can be extended to benefit similar health environments, such as extending the monitoring of the use of the application and the booklet for 6 months depending on the availability of participants and financial resources, since, currently, these factors are also limitations of this study.

## Figures and Tables

**Table 1:** Inclusion and exclusion criteria for RCT participants.

Inclusion Criteria	Exclusion Criteria
Be aged between 60 and 79 years old	Diagnosed for DM 1
Diagnosed with DM 2 or pre-diabetes	Use insulin
Be literate	Having chronic complications of DM (renal failure, retinopathy, neuropathy, limb amputation and diabetic foot)
Have a cell phone with access to an instant messaging application (WhatsApp) *	Have mobility limitations (Timed Up-and-Go-TUG > 20 s)
Have independence and autonomy in daily life activities	Present moderate/severe cognitive impairment (Mini-Cog < 3)
Being independent for activities of daily living or needing adaptation or supervision from third parties to carry out activities of daily living.	present high blood pressure-stage 3-PA 180/110
Be available to attend appointments	Participating in another clinical trial
	Present diagnosis of progressive neurological disease or other medical condition that prevents exercise

* Access to WhatsApp is needed because older adults in Brazil predominantly use this application. In this sense, the communication between researchers and the participants of the intervention group and the control group regarding the stages of the research will take place through this tool [34,35].

**Table 2:** Interfaces and sections of the application and educational booklet.

CID—Design and Development	
App (Intervention Group)	Booklet (Control Group)
User profile: name, age, gender, weight, height BMI	Weight control, physical exercise, healthy eating and medication use records
Concepts related to DM 2: Crossword Puzzle	Concepts related to DM 2 Foot care guidelines
Healthy eating guidelines: word search game	General guidelines on healthy eating: word search game
Food diary and educational messages about healthy eating	General guidelines on healthy eating
Aerobic and resistance exercise protocol	Guidelines on aerobic and resistance exercise: “Find the difference game”
Registration of continuous use medications and reminder alarm	Guidelines for medication adherence
Guided meditation and self-massage	Encouragement for controlling stress, smoking and alcoholism

## Data Availability

The raw data supporting the conclusions of this article will be made available by the authors on request.
